# The effect of different poly fibers separator-modified materials on blocking polysulfides for high performance Li-S batteries

**DOI:** 10.3389/fchem.2022.931201

**Published:** 2022-08-11

**Authors:** Ling Meng, Zhaoxia Sun, Guanghang Sun, Xiting Zhang, Meng Dan, Jin Long, Jian Hu

**Affiliations:** ^1^ Huangpu Hydrogen Innovation Center/Guangzhou Key Laboratory for Clean Energy and Materials, School of Chemistry and Chemical Engineering, Guangzhou University, Guangzhou, China; ^2^ National Engineering Research Center of Paper-making and Pollution Control, School of Light Industry and Engineering, South China University of Technology, Guangzhou, China

**Keywords:** poly (p-phenylene terephthalamide), poly (p-phenylene benzobisoxazole), lithium–sulfur battery, separator, shuttle effect

## Abstract

Herein, we reported that KOH impregnation can generate a large number of porous structures with fruitful nitrogen self-doped groups during the carbonized process for poly (*p*-phenylene terephthalamide) fiber and poly (*p*-phenylene benzobisoxazole) fiber (denoted as PPTA and PBO, respectively). The intrinsical insulation, volume change, and shuttle effect of polysulfides then can be more significantly improved for the PBO-coated separator than the PPTA case. The discharge capacity primary achieves 1,322 mA h/g, which retains 827 mA h/g even after 200 cycles at 0.2 C for the cell with PBO-coated separator. The reversible specific discharge capacity maintains 841 mA h/g with a Coulomb efficiency of 99.7% at 5 C. The nitrogen self-doped nanocarbon particles are etched by KOH with the simple one-step preparation, which has promising application as Li-S battery cathode.

## Introduction

Li–S batteries, as an alternative to traditional lithium-ion batteries, have large energy density (2,600 Wh/kg) and high specific capacity (1,675 mA h/g) (Hou et al., 2021) and can be regarded. However, the LSBs should overcome the following obstacles which will rapidly degrade battery performance (Yang et al., 2019; Hou et al., 2021), such as electronically and ionically insulation of S, large volume change (∼80%) between the charged state (elemental sulfur) and the discharged state (lithium sulfide) during cycling (Wu et al., 2020), as well as shuttle effect induced by the high solubility and mobility of the lithium polysulfide (LiPS) intermediates. Repeated volume changes tend to damage the conductive structure of the electrode (Yang et al., 2019). The “shuttle effect” of polysulfides leads to loss of sulfur-containing components in the battery (Li and Zhang, 2020).

The high-porosity nanofibers with a large specific surface area can enhance electronic/ionic conductivity, endure volumetric change, and physically trap LiPSs (Yin et al., 2013; Huang et al., 2015; Zhu et al., 2021). However, the interaction between polar LiPSs and nonpolar carbon is weak, leading to LiPS effusion and loss from the cathodes and thus rapid capacity fading during cycling. Carbon hosts with heteroatom doping (e.g., N, O, S, P) can increase polarity, which favors physically LiPS trapping and improves the LiPS conversion into solid Li_2_S (Vázquez-Santos et al., 2012a; Zhang et al., 2012; Li et al., 2017; Han et al., 2021; Yao et al., 2021). Metallic Co (Moorthy et al., 2019; Jin et al., 2021; Luo et al., 2021), Fe (Sun Y et al., 2019), Ti (Ni et al., 2020; Yao et al., 2021), Se (Luo et al., 2021), Ni (Jin et al., 2021), Mo (Li et al., 2021a; Kong et al., 2021), etc doped on the porous carbon modified separators also can improve the electrochemical performance of lithium–sulfur batteries. Therefore, three-dimensional (3D) nanocarbon materials can act as S hosts with shorter ionic/electronic transport distances and more active sites.

**SCHEME 1:**
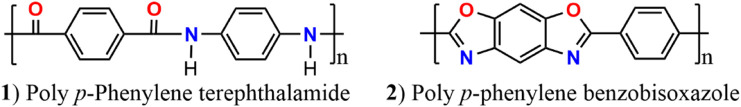
Structures of PPTA and PBO.

In this work, the poly (*p*-phenylene terephthalamide) and poly (*p*-phenylene benzobisoxazole) (abbreviated as PPTA and P BO, respectively) are high-performance fibers (see Scheme 1) with high strength and modulus and excellent heat/corrosion resistance (Zhang et al., 2012; Yang et al., 2019). The nitrogen-containing hierarchical 3D poly fibers with rich porous structure are based on a new KOH–assisted tactic, which is different from the H_3_PO_4_–activated PBO steel rod shape fiber as previously reported (Vázquez-Santos et al., 2012a; Li et al., 2017). The self-doped N porous carbon, prepared by a simple and fast method, was used as a separator modification layer in lithium–sulfur batteries with a high N retention rate. The rich porous structure can limit the lithium polysulfide from going to the positive electrode area. The N elements generated *in situ*, which are evenly distributed in the hierarchical porous carbon material, can improve the chemical adsorption of polysulfide. Large specific surface area can tolerate the S volume expansion during lithiation. Our 3D hierarchical poly-fiber structures also can act as conductive networks and integral structure supporters. It was found that the capacity attenuation rate is only 37.4% after 200 cycles at 0.2°C. The reversible specific capacity maintains 841 mA h/g with 99.7% Coulomb efficiency during the discharge process at 5 C. The modified separator restrains efficiently the LiPS shuttle and thus improves significantly the electrochemical kinetics of Li-S batteries (Li et al., 2021b; Yao et al., 2021). This work opens up new opportunities for the construction and design of hierarchical three-dimensional (3D) poly fiber separators for high-performance Li-S batteries application (Li et al., 2021b; Han et al., 2021; Yao et al., 2021).

## Experimental

### Materials

From China: 99.95% sulfur (Aladdin); lithium (KeJing); N, N-dimethylformamide (DMF, Aladdin); electrolyte (1.0 M DOL/DME LiTFSI with 1% LiNO_3_, DuoDuo); KOH (Analytically pure, GuangZhou Chemical).

From Japan: PPTA fiber (1,080, Diameter:12 μm, Length: 6 mm, Teijin); PBO fiber (Zylon^®^ AS, Diameter: 13 μm, Length: 6 mm, Toyobo); Ketjen black (ECP600JD, Lion).

From France: polyvinylidene fluoride (PVDF, ARKEMA, HSV900).

### Preparation of microfiber hand sheets based on PPTA or PBO

PPTA micro/nanofibers were fibrillated by the beating process in a pulp refiner (AWE14, AIFUDE, China). After the gap between rotor and stator disks of the refiner was adjusted with 9.9, 2.6, 1.7, 1.1, 0.5, 0.4, and 0.2 mm, we can obtain various degrees of PPTA fibrillation. The PPTA nanofiber was prepared by a classical fibrillation process according to our previous report (Meng et al., 2021). PBO fibers were fibrillated by using a Walli beater (NO.2505, KRK, Japan) to obtain various degrees of fibrillation with control of pressure, pulp concentration, flow velocity, and beating time. The most important factor is the beating time. PPTA or PBO was stirred for 5000r by HR2101 (Netherlands) and dispersed in deionized water. Generally, PPTA and PBO papers were further filtrated on Automatic Sheet Former (Kumagai Riki Kogyo CO., Ltd) to produce wet hand sheets, which can be dried at 105°C and held for 15 min using a plate dryer (No.140, EMERSON, American). Two kinds of high-performance microfiber paper-based materials can be obtained, which were labeled as PPTA-P and PBO-P, respectively.

### Activation with KOH and carbonization of PPTA and PBO handsheets

The PPTA- or PBO paper did not need the pre-oxidation progress in the present work, which is significantly different from those in the previous report (Vázquez-Santos et al., 2012b; Li et al., 2017). They are directly immersed in 25% KOH solution with the mass ratio of 1:1 for 10 h. After drying, the impregnated PPTA- or PBO-paper was heated with a rate of 5 C min^−1^ until at 800 C and held for 1 h under an N_2_ atmosphere (flow rate: 200 ml/min) in a tubular furnace. The hand sheet then goes through natural cooling, neutralization with 1 mol L^−1^ hydrochloric acid, rinsing with a large amount of deionized water, and drying for later use. Two kinds of hand sheets with KOH activation and carbonization were labeled as PPTA-KOH and PBO-KOH.

### Preparation of PPTA and PBO coated separators

**FIGURE 1:**
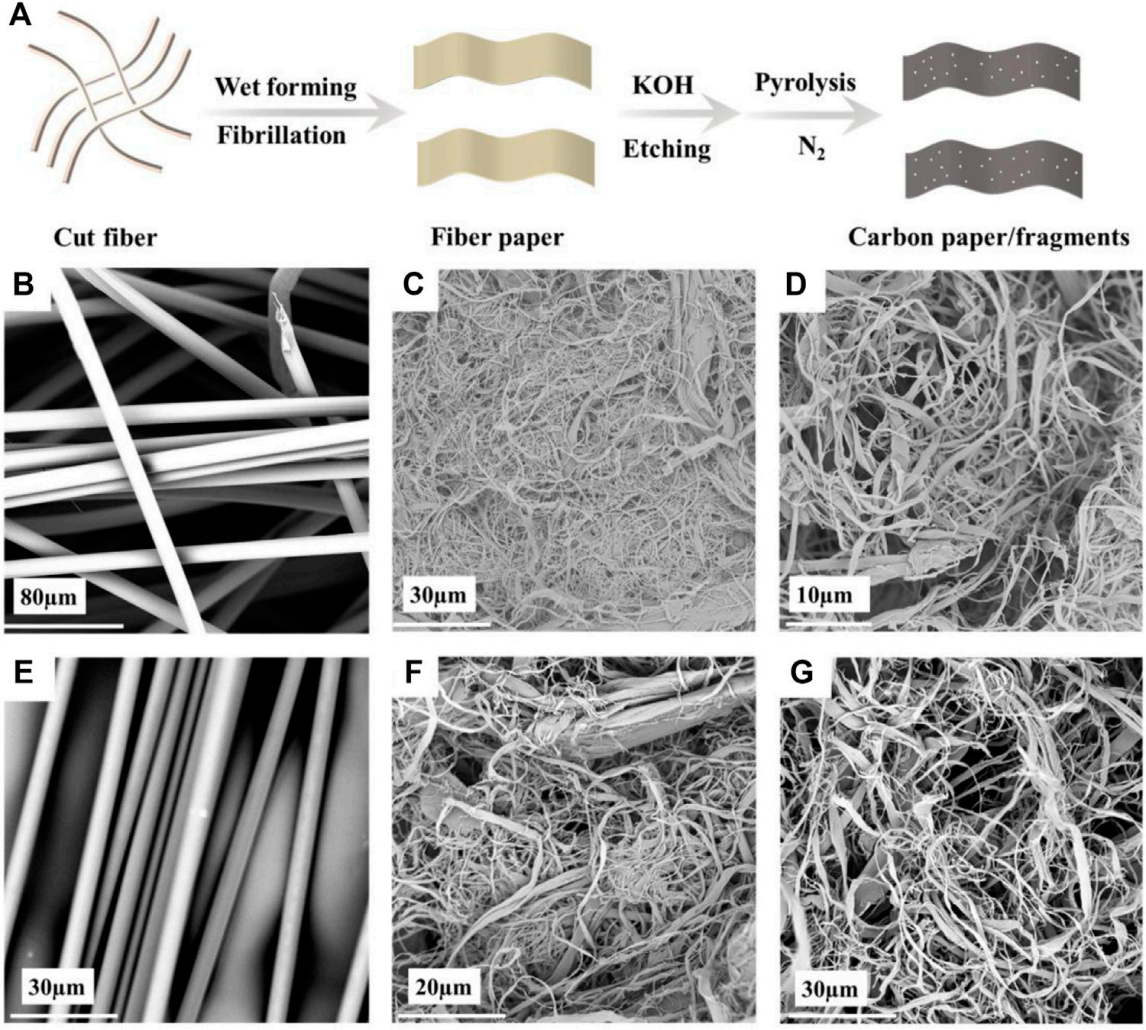
**(A)** Process of preparation of PPTA and PBO nano carbon particles. Scanning electron microscope (SEM) images of **(B)** PPTA short cut fiber, **(C)** PPTA microfiber, **(D)** PPTA paper, **(E)** PBO short cut fiber, **(F)** PBO microfiber, and **(G)** PBO paper.

After ball milling PPTA–KOH and PBO-KOH for 10 and 16 h, respectively, the particle size reaches the same micro-nano level. Figure 1A shows the preparation process of PPTA and PBO nano carbon particles. The carbon particles and PVDF were mixed in a ratio of 9:1, and then an appropriate amount of NMP was added dropwise to adjust the viscosity and quickly ground and then coated on the Celgard 2500 PP separator with a thickness of 50 µm. The two kinds of samples after ball milling were labeled as PPTA-KOH-G and PBO-KOH-G, and the two kinds of coated separators were labeled as PPTA-KOH-S and PBO-KOH-S, respectively.

### Characterization

The energy-dispersive spectroscopy (EDS) was used with X-MaxN20 (Oxford) to characterize element distribution and field emission scanning electron microscopy (FESEM, Merlin, Zeiss) was performed to probe morphology. The four-probe method was used to measure conductivity. LabRAM Aramis can export Raman spectra. PANalytical X’pert Powder can provide X-ray diffraction patterns. The Axis Ultra DLD spectrometer can provide X-ray photoelectron spectroscopy (XPS). The Autosorb-IQ2-MP (Quantachrom, American) was used to measure specific surface area and pore volume according to N_2_ adsorption/desorption isotherms. JEM2100F (JEOL, Japan) was applied to test the transmission electron microscope (TEM) image.

### Electrochemical analysis

Sulfur slurry was prepared by wt% mixture of sulfur (70), ketjen black (20), and polyvinylidene fluoride (10) in N-methyl pyrrolidinone solvent, which then was cast onto carbon-coated aluminum foil and dried for 12 h at 50 C. The sulfur has a mass loading of about 3.8 mg/cm^2^ in the cathode. The glove box was filled with 2032 batteries in an argon atmosphere. The electrolyte was about 40 μl, which comprised 1 M LiTFSI and 0.1 M LiNO_3_. In addition, 1, 2-dimethoxyethane (DME) and 1, 3-dioxacyclopentane (DOL) were also added to the electrolyte with a volume ratio of 1:1.

LAND CT2001A was used to record the galvanostatic charge/discharge for battery testing with a potential range from 1.7 to 2.6 V. The electrochemical workstation CHI604E can collect cyclic voltammograms (CV) by scanning potential from 1.5 to 3.0 V with the rate of 0.1 mV/s and electrochemical impedance spectroscopy (EIS) fixed at an amplitude of 5 mV with 10^−2^–10^5^ Hz frequency range.

## Results and discussion

### Characterization of PPTA-based and PBO-based carbon coating

Figure 1A displays the process of preparation of PPTA- or PBO-based carbon coating. PPTA and PBO are smooth surfaces, typical skin-care structures, and high-performance fibers (Figures 1B,E). After exerting mechanical force, the tough skin layer was destroyed (Figures 1C,F) and the fiber was split longitudinally, which promotes fiber separation to produce the microfiber. In the beating process, the friction of fiber-cutter or fiber-fiber facilitates micro-fiber bundles to separate from the backbone of fibers. Figures 1D,G is the paper after wet forming.

**FIGURE 2:**
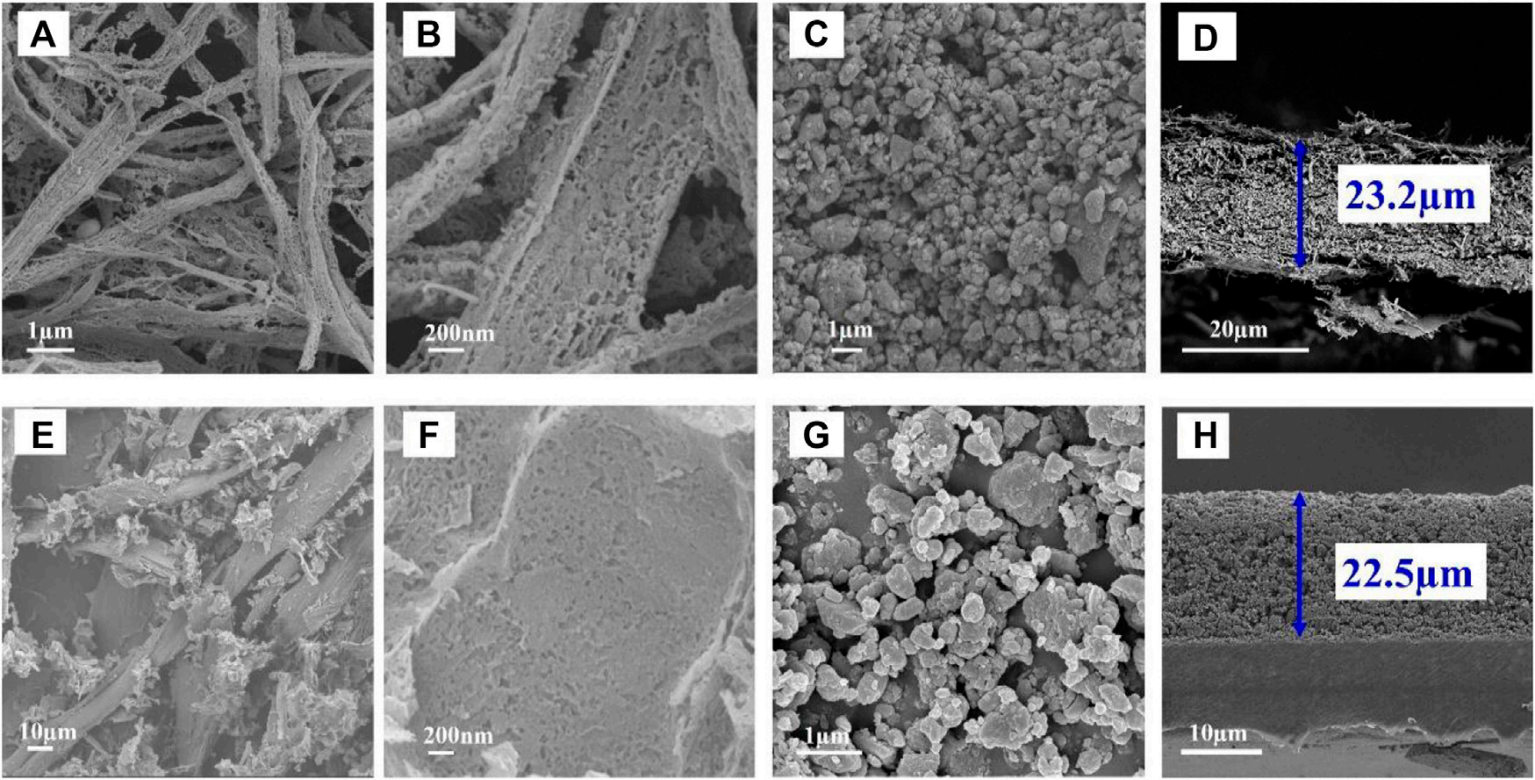
SEM images of **(A,B)** PPTA-KOH, **(C)** PPTA-KOH particles after ball milling; **(D)** the cross-section of PPTA coated separator; SEM images of **(E,F)** PBO-KOH, **(G)** PBO-KOH particles after ball milling; **(H)** the cross section of PBO coated separator.

Figures 2A,B show the SEM images of carbonized PPTA after KOH impregnation, in which the fibers are covered with abundant holes. Furthermore, the holes are larger and shallower for the PBO case (see Figures 2E,F, SEM images). Figures 2C,GFigures 2c, and 2g indicate the particles have a micro-nano size after ball milling of PPTA-KOH and PBO-KOH, respectively. The thicknesses are 23.2 and 22.5 μm for PPTA-KOH-S (see Figure 2D) and PBO-KOH-S (see Figure 2H), respectively.

Supplementary Figure S1 shows the C, N, and O mapping of PPTA-KOH-G and PBO-KOH-G. Supplementary Table S1 shows the weight percent of C, N, H, and S elements in PPTA-P、PPTA-KOH-G、PBO-P, and PBO-KOH-G. The N content of PPTA-KOH-G and PBO-KOH-G is 4.74 and 5.50 wt%, respectively.

Supplementary Figure S2 and Supplementary Table S2 reveal that the PBO fibrillated fiber nano-carbon material (denoted as PBO-KOH-G) has a high specific surface area (2,104 m^2^/g) and total pore volume (1.3 cm^3^/g), which favors electrolyte penetration and polysulfide adsorption as compared with PPTA-KOH-G (1,677 m^2^/g and 1.2 cm^3^/g, respectively). Both Supplementary Figures S2B,D displayed type IV isotherms with an adsorption system of H4-type hysteresis loop corresponding to slit-like pores, which display mesopores and micropores coexisted for both PBO-KOH and PPTA-KOH. PBO-KOH has a higher proportion of micropores and larger pore volume than PPTA-KOH. The surface force relative to the pore wall plays a dominant role in micropore adsorption, while the surface force together with capillary force is significant in mesopore adsorption.

Generally, carbon materials have two characteristic G (1,600 cm^−1^) and D (1,360 cm^−1^) peaks for sp^2^ atoms in Raman spectra, which represent the bond stretching of chains and rings and the breathing modes of rings (structural imperfection) (Wu et al., 2019), respectively. The I_D_/I_G_ value is smaller for PBO-KOH-G (0.994) than that (1.051) for PPTA-KOH-G, which indicated the higher graphitization degree of PBO-KOH-G (Zhu et al., 2019).

Supplementary Figure S3 shows the TEM of PPTA-KOH-G and PBO-KOH-G. It can be seen from the TEM images that both PBO-KOH and PPTA-KOH present an amorphous carbon structure without obvious lattice fringes. Among them, PPTA-KOH has some black spots with different light and dark, which should be related to the precursor.

**FIGURE 3:**
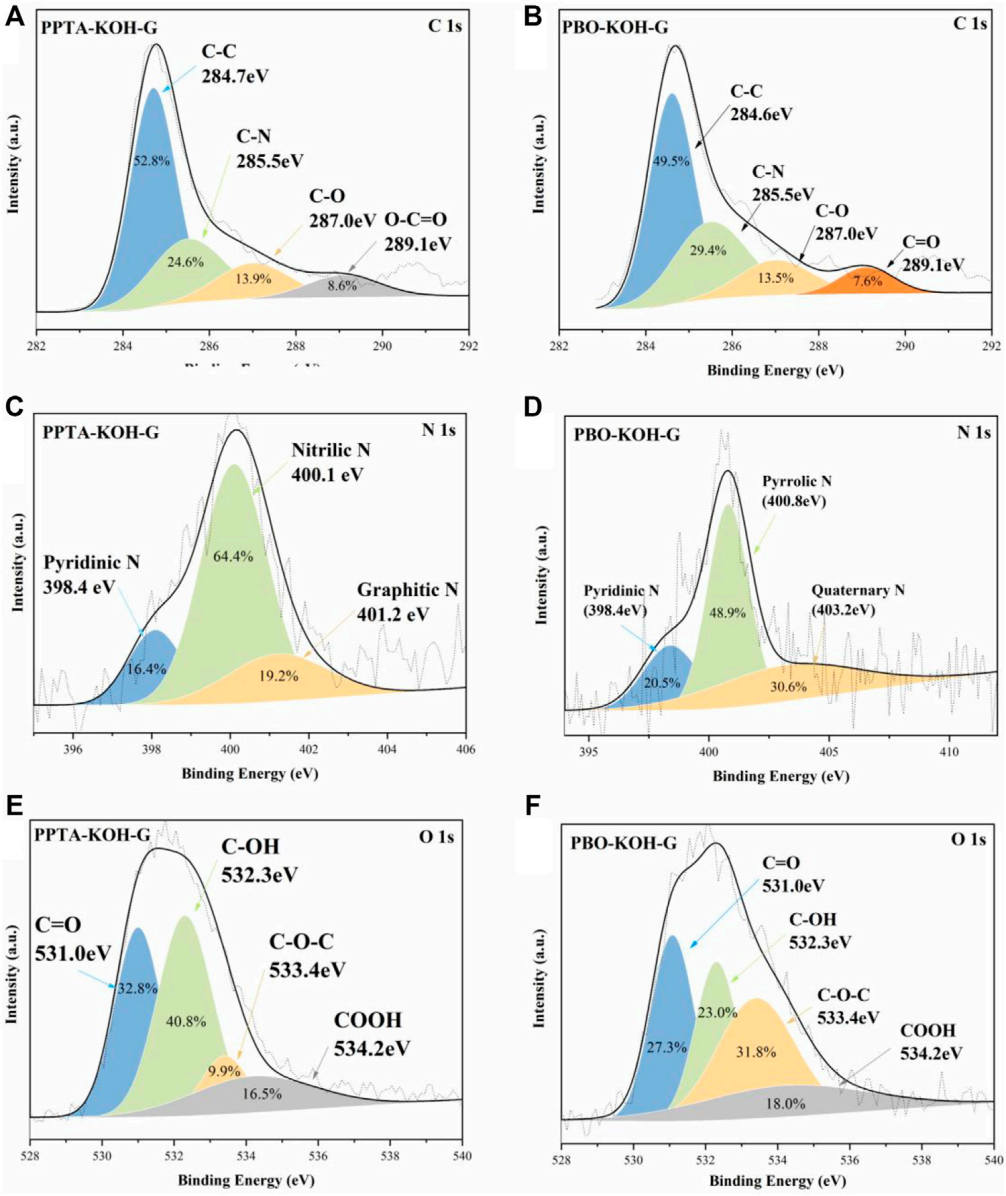
High-resolution XPS spectra of C 1s **(A)** and **(B)**; N 1s **(C)** and **(D)**; O 1s **(E)** and **(F)** for PPTA-KOH-G and PBO-KOH-G, respectively.

The chemical composition of the carbon materials can be investigated by the XPS spectrum, which can be divided into four peaks, namely, C-C (284.7 eV), C-N (285.5 eV), C-O (287.0 eV), and C=O (289.1 eV) (Figures 3A,B). It should be noted that three nitrogen doping forms (pyrrolic N, pyridinic N, and Quaternary N) are retained even at a carbonization temperature of 800°C. Therefore, N components are chemically stable, which can produce more polar sites to immobilize polysulfide intermediates in carbon papers (Qian et al., 2019). Figure 3C displayed N 1s peaks in PPTA-KOH-G. The high-resolution spectra exhibit fine split signals for pyridinic, nitric, and graphitic N at 398.4, 400.1, and 401.2 eV, respectively. The pyridinic N is formed with a six-membered ring at the edges or defects while graphitic N replaces the C atom of a hexagonal ring in the graphitic lattice (Saroha et al., 2021). Figure 3D displays high-resolution N (1s) XPS spectrum which can be divided into three peaks at 398.4 (pyridine N), 400.8 (pyrrole N), and 403.2 eV (quaternary amine N) for PBO-KOH-G, respectively. The pyridine N (sp^2^) atom connected to two carbon atoms with the six-membered ring, is usually located at the edge or defect site of the graphite layer (Sun Z et al., 2019). The lone-paired electrons of the N atom do not participate in the π conjugate system. The pyrrole has the other N (sp^2^) atom in the five-membered ring (Han et al., 2021), in which the lone-paired electrons participate in the π conjugate system, leading to rich electron cloud density (Wang et al., 2020). Quaternary amine N belongs to chemical N, while pyrrolic and pyridinic N were attributed to structure N (Song et al., 2014). The oxygen atoms also can immobilize the polysulfide intermediates in the carbon network (Li et al., 2021b), which are presented as hydroxyl and carboxylic groups in the aromatic graphene network (Li et al., 2021c). Oxygen-containing groups, especially ether and carbonyl groups, are electrochemically active, which can improve the electrochemical performance of carbon materials (Xiao et al., 2019). The O 1s high-resolution XPS spectrums are displayed in Figures 3E,F for PPTA-KOH-G and PBO-KOH-G, which can be divided into four peaks of carboxyl group (-COOH, 534.2 eV), ether group (-CO-C, −533.4 eV), hydroxyl group (-C-OH, 532.3 eV), and carbonyl group (-C=O, 531.0 eV). The C=O and C-OH peaks are stronger than those of COOH and C-O-C. It should be noted that the areas of these peaks decrease except for the COOH group after activation.

### Electrochemical evaluation

**FIGURE 4:**
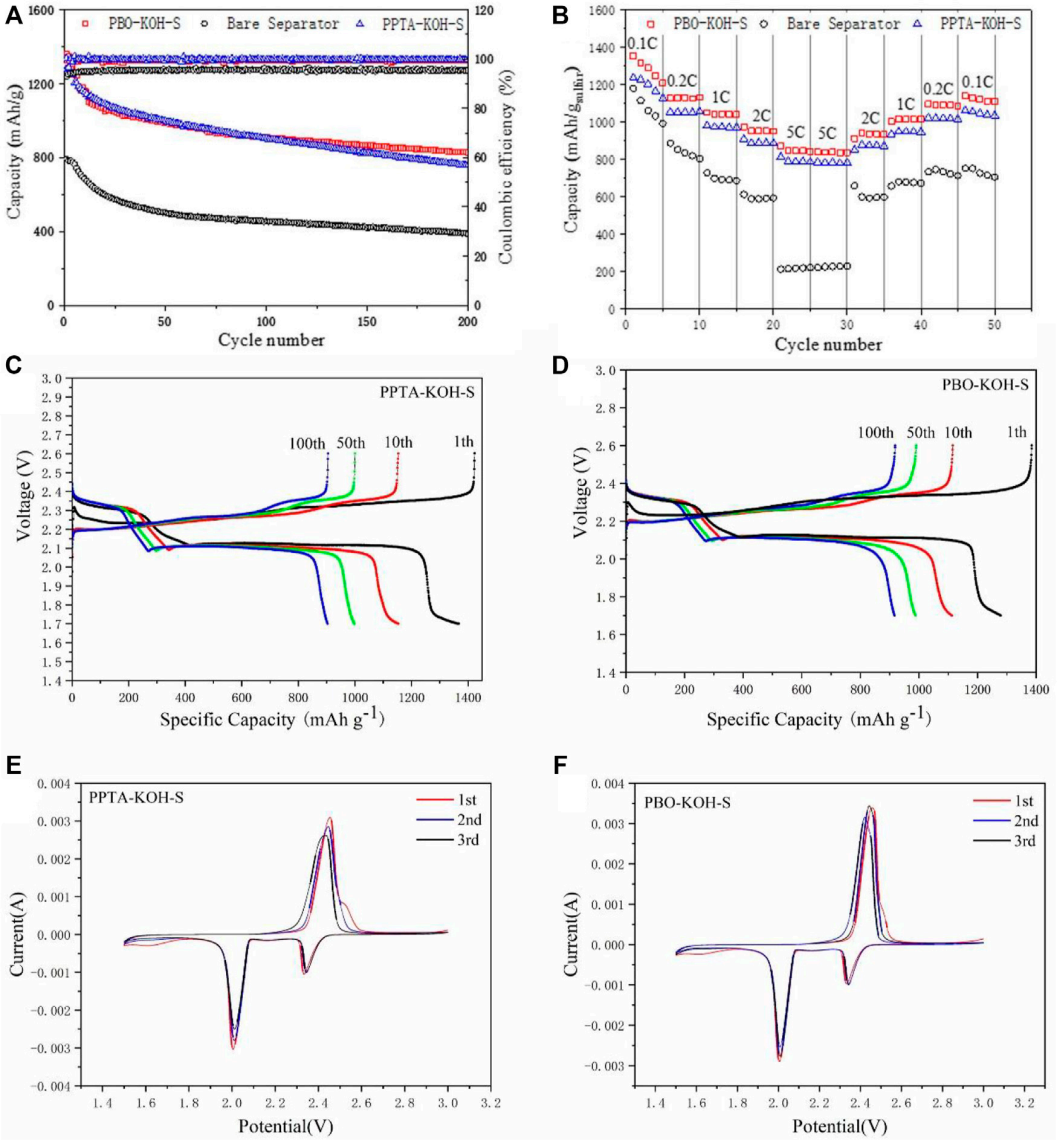
**(A)** Charging and discharging curves of PPTA-KOH-S and PBO-KOH-S Li-S batteries at 0.2 C by 200-cycle test; **(B)** Rate performance of Li-S batteries with PPTA-KOH-S and PBO-KOH-S at 0.1, 0.2, 0.5, 1, 2 and 5 C; The charging and discharging curve for 1st, 10th, 50th, 100th of Li-S batteries with **(C)** PPTA-KOH-S and **(D)** PBO-KOH-S; The CV curve of Li-S batteries with **(E)** PPTA-KOH-S and **(F)** PBO-KOH-S.

The cycling performances are illustrated in Figure 4A with PPTA-KOH-S and PBO-KOH-S as separators, in which the initial capacities are 1,339 and 1,322 mA h/g for the cells, respectively. PBO-KOH-S displays high reversible discharge capacities of 827 mA h/g by 200-cycle test as compared with PPTA-KOH-S. Moreover, all batteries have nearly 100% Coulombic efficiencies, which demonstrates that PBO-KOH-S separators can effectively inhibit the sulfur shuttle effect. PBO-KOH-S also exhibits better rate performance at current densities with the range from 0.1 to 5 C (Figure 4B) than PPTA-KOH-S. The battery capacities of PBO-KOH-S decline gradually from an initial 1,352.9 to 1,127.7, 1,050.5, 973, 870.8, and 840.6 mA h/g. The battery capacity of PBO-KOH-S was recovered to 1,108.7 mA h/g as the current density is adjusted back to 0.1 C. The PBO-KOH-S battery continues to discharge steadily. The above facts prove high reversibility for PBO-KOH-S as compared with PPTA-KOH-S batteries, which are mainly derived from micro- and mesopores differences between PBO-KOH-S and PPTA-KOH-S.

Generally, the electrochemical performance of Li-S batteries with PBO-KOH-S is as excellent as those of Li-S batteries with other separators, such as rGO/Co-Ni-S (Wu et al., 2022), biomass carbon based on N, O co-doped chlorella (Jin et al., 2021) and Co-N-C hollow nanocages (Jin et al., 2021), especially under the current density of 5 C (see Supplementary Table S3).

Figures 4C,D show typical charge–discharge voltage profiles from 1.7 to 2.6 V for cells at 0.2 C with different separators. The first plateau in the discharge curve can be attributed to the process of sulfur element reduced to soluble polysulfides at 2.3 V, and the second can be assigned to soluble polysulfide reduction followed by the generation of insoluble Li_2_S_2_/Li_2_S at 2.1 V. Therefore, PBO-KOH-S cell performs stronger redox kinetics for polysulfide conversion due to micro- and mesopores with high volume than PPTA-KOH-S cell.

The redox mechanism was investigated by the CV tests in which coin-type cells were scanned from potential 1.5 to 3.0 V at a rate of 0.1 mV/s. Two main peaks at 2.30 and 2.02 V (see Figures 4E,F) were detected corresponding to reduction from sulfur element to higher-ordered polysulfides (Li_2_S_n_, 4 ≤ n ≤ 8) and further to low-ordered sulfides (Li_2_S_2_, Li_2_S) in the cathodic scan (curves below) (Moorthy et al., 2019), respectively. However, only one oxidation peak at 2.38∼2.44 V can be detected due to the transformation from low-to higher-order sulfides and finally generates elemental sulfur in the anodic scan (see curves above) (Cheng et al., 2021).

In comparison to PPTA-KOH-S (Figure 4E), the anodic/cathodic peaks indicate substantially mitigated polarization and hence improve electrochemical reduction for PBO-KOH-S (Figure 4F). Furthermore, the altitude of peaks maintains almost identical by the 3-cycle test, demonstrating that the cell with PBO-KOH-S has good cycling stability. Two redox peaks of PBO-KOH-S have unsharp shapes and low peak currents, which proves the high reactive kinetics of Li-S cells from soluble polysulfides to Li_2_S/Li_2_S_2_. Therefore, the results illustrate that the electrochemical polarization can be effectively reduced, leading to improved kinetics and electrical conductivity in PBO-KOH-S.

EIS of the cells is conducted to understand for more detail in the better cell performance of PBO-KOH-S than PPTA-KOH-S (see Supplementary Figure S4). The EIS profile consists of the single semicircle and inclined line for PPTA-KOH-S while two semicircles and an oblique line for PBO-KOH-S after 100 cycles at 0.2C.

Supplementary Figure S5A shows the SEM of PPTA-KOH-S after 200 cycles. It can be seen that the polysulfide clusters on the carbon particles, which have been completely fused with the PPTA particles on the separator and are evenly distributed. The PPTA carbon material can not only provide an effective conductive path as a conductive material but also serve as an adsorption layer and a barrier layer to inhibit polysulfides. Supplementary Figure S5B is the SEM of the recycled PBO-KOH-S porous carbon coating layer. The polysulfide is firmly adsorbed in the pores, and the physical confinement inhibits expansion and shuttle (Song et al., 2014; Schüpfer et al., 2021). This kind of “filtration” method uses the functional modification layer to remove the polysulfide. The material is trapped on the side of the separator near the positive electrode. Supplementary Figures S5C,D show the S of PPTA-KOH-S and PBO-KOH-S after cycling, respectively. From the UV-visible spectrum of Supplementary Figure S5E, it can be known that the absorption peak intensity of the solution after PBO-KOH-G adsorbed lithium polysulfide is the lowest, which further proves that PBO-activated carbon has a stronger adsorption capacity for polysulfide (Hou et al., 2017). From Supplementary Figure S5F, it can be clearly seen that the color of PBO-KOH-G is lighter, indicating that its ability to adsorb polysulfides is stronger (Yang et al., 2021). In addition, the XPS measurement was also conducted to study the strong chemical adsorption of polysulfide of PBO-KOH-G after adsorption of polysulfide, as shown in Supplementary Figures S7A–C. The result showed that the binding energy of C 1s, N 1s, and O 1s for the PBO-KOH-G sample after adsorption of polysulfide showed obvious changes as compared with the PBO-KOH-G sample before adsorption of polysulfide (Supplementary Figure S6), indicating that the PBO activated carbon with a stronger adsorption capacity for polysulfide. Meanwhile, the existence of polysulfide also is proved by the S 2p XPS spectra result (Supplementary Figure S7D).

## Conclusion

In this work, KOH impregnation can efficiently generate mesopores. Furthermore, PBO exhibits better cycle and rate performance than PPTA. The nitrogen self-doped mesoporous physically inhibits the Li_2_S_n_ diffusion or shuttle across the separator and enhances the chemical adsorption of polysulfides, thereby improving the electrochemical performance of the Li-S battery. The porous carbon nanocarbon particles are light and uniform with a load per unit area of <0.08 mg/cm^2^. The porous structure can act as buffer space for volume changes during the electrode cycle. For PBO functional layer, the maximum specific surface area is 2,104 m^2^/g and the maximum pore volume is 1.29 cm^3^/g; The specific capacity decrease from 1,322 to 827 mA h/g after 200 cycles of cycling so that the capacity attenuation rate is only 37.4% with the single-lap attenuation rate of 0.187% at a current density of 0.2 C. All in all, this work provides a tandem strategy for facilitating hierarchical three-dimensional (3D) poly fibers separator toward high-performance Li-S batteries.

## Data Availability

The original contributions presented in the study are included in the article/Supplementary Material; further inquiries can be directed to the corresponding authors.
