# Supplementary material for: The effect of different poly fibers separator-modified materials on blocking polysulfides for high performance Li-S batteries
Source: Front Chem. 2022 Aug 11;10:931201. doi: 10.3389/fchem.2022.931201 (PMC9403007; doi:10.3389/fchem.2022.931201)
Supplement: Supplementary file 1 [file DataSheet1.docx]

**Supporting Information**

**The Effect of Different Poly Fibers Separator-Modified Materials on Blocking Polysulfides for High Performance Li-S Batteries**

Ling Meng^1^, Zhaoxia Sun^2^, Guanghang Sun^2^, Xiting Zhang^1*^, Meng Dan^1^, Jin Long^2*^, Jian Hu^2^

1. Huangpu Hydrogen Innovation Center/Guangzhou Key Laboratory for Clean Energy and Materials, School of Chemistry and Chemical Engineering, Guangzhou University, Guangzhou 510006, PR China.
2. National Engineering Research Center of Paper-making and Pollution Control, School of Light Industry and Engineering, South China University of Technology, Guangzhou, 510640, China

*Corresponding author. E-mail address:zhxt@gzhu.edu.cn^1^,longjin@scut.edu.cn^2^

Current address:

1. Huangpu Hydrogen Innovation Center/Guangzhou Key Laboratory for Clean Energy and Materials, School of Chemistry and Chemical Engineering, Guangzhou University, Guangzhou 510006, PR China.
2. Current address: National Engineering Research Center of Paper-making and Pollution Control, School of Light Industry and Engineering, South China University of Technology, Guangzhou, 510640, China

**Table of contents**

| **Section 1** | The SEM images of cross-sectional (a)and the surface (b)of sulfur cathode, mapping of C(c) and S(d). | Figure *S1* |
| --- | --- | --- |
| **Section 2** | The mapping of (a) C, (b) N, and (c) O of PPTA-KOH-G and the mapping of (d) C, (e) N, and (f) O of PBO-KOH-G. | Figure *S2* |
| **Section 3** | The (a) N_2_ adsorption/desorption isotherms and (b) pore size distributions of PPTA-KOH-G, (c) N_2_ adsorption/desorption isotherms and (d) pore size distribution of PBO-KOH-G | Figure *S3* |
| **Section 4** | The TEM of (a) (b) PPTA-KOH-G and(c) (d) PBO-KOH-G. | Figure *S4* |
| **Section 5** | The EIS profiles of Li-S cells with PPTA-KOH-S and PBO-KOH-S as separators after 100 cycles and equivalent circuit models. | Figure *S5* |
| **Section 6** | The EIS profiles of Li-S cells with PPTA-KOH-S and PBO-KOH-S as separators after 100 cycles and equivalent circuit models. | Figure *S6* |
| **Section 7** | The (a) C 1s, (b) N 1s, (c) O 1s, and (d) S2p XPS spectra of PBO-KOH-G after adsorption of polysulfide | Figure *S7* |
| **Section 8** | The weight percent of C, N, H and S element in PPTA-P、PPTA-KOH-G、PBO-P and PBO-KOH-G. | Table *S1* |
| **Section 9** | Pore analysis of PPTA and PBO based carbon materials. | Table *S2* |
| **Section 10** | Summary of the electrochemical properties of Li-S batteries with various coating separators. | Table *S3* |
| **Section 11** | Atomic compositions of the C、N、O and S measured by XPS survey results after cycled | Table *S4* |


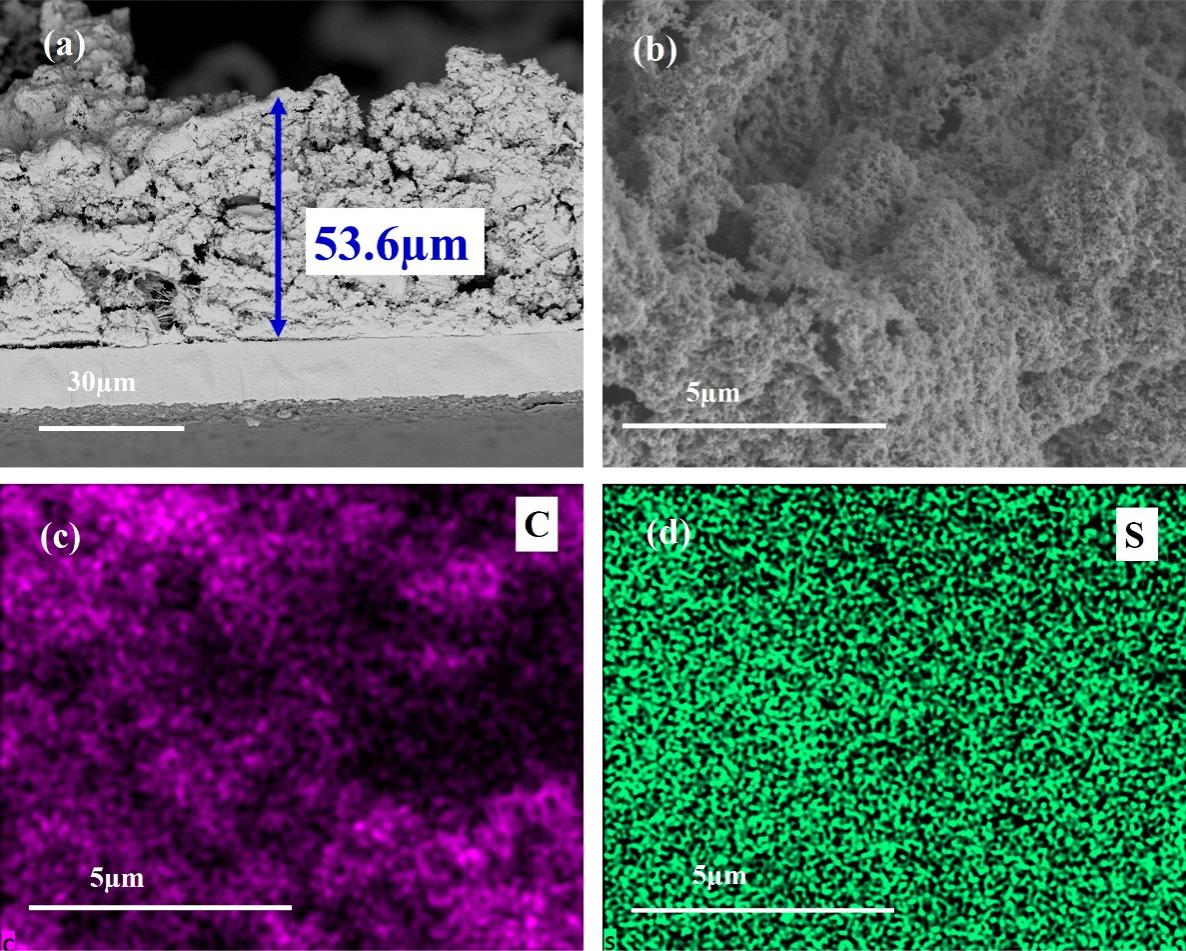
 **Figure S1.** The SEM images of cross-sectional (a)and the surface (b)of sulfur cathode, mapping of C(c) and S(d).


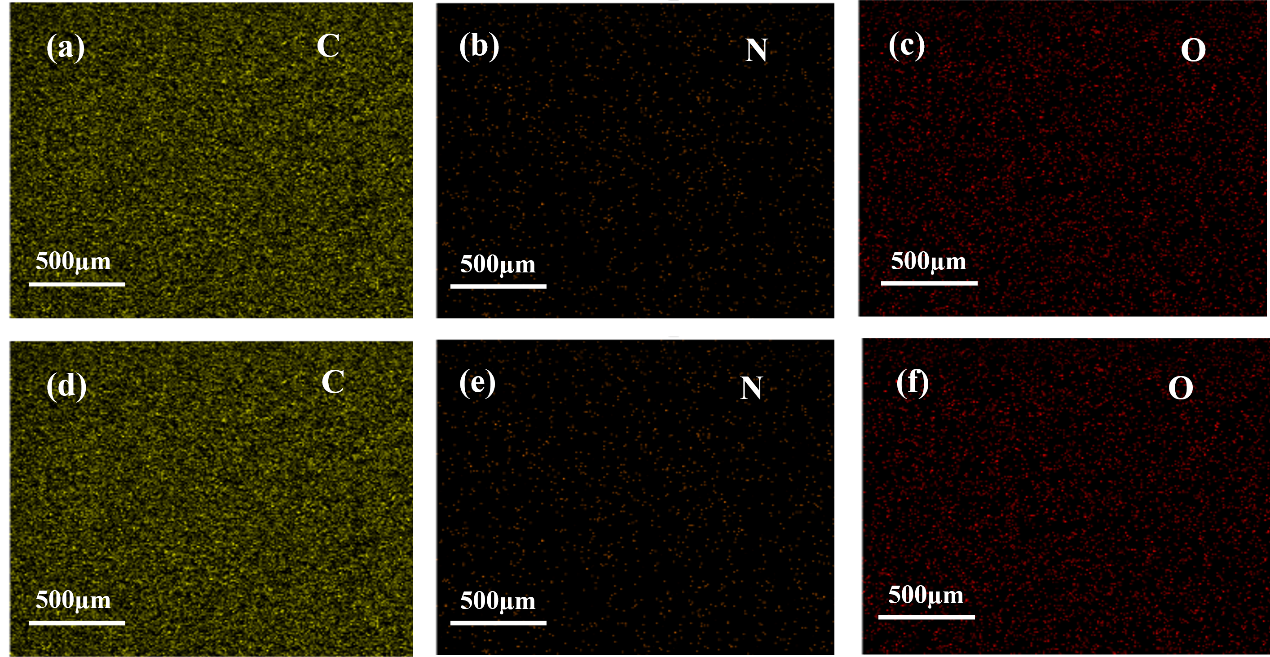


**Figure S2.** The mapping of (a) C, (b) N, and (c) O of PPTA-KOH-G and the mapping of (d) C, (e) N, and (f) O of PBO-KOH-G.


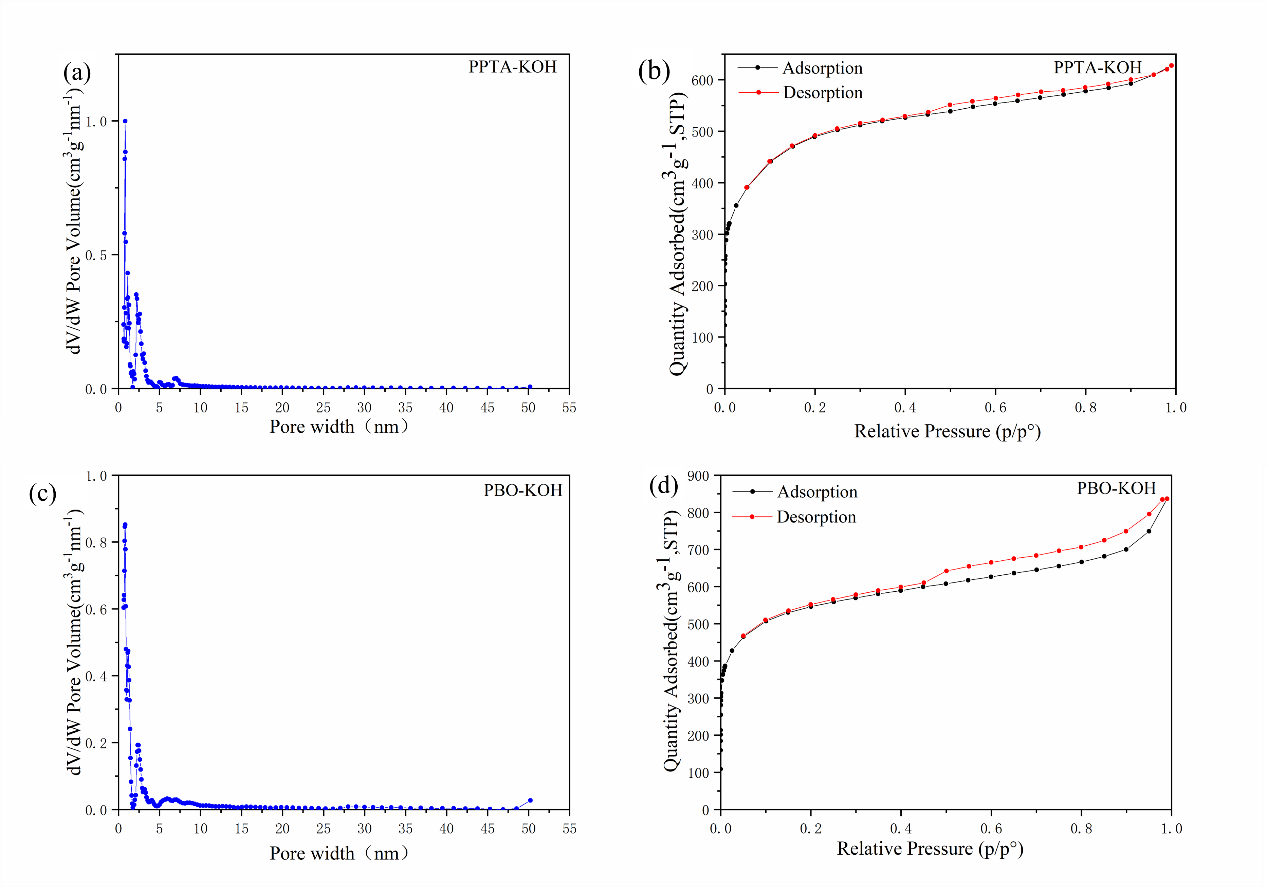


**Figure S3.** (a) N_2_ adsorption/desorption isotherms and (b) pore size distributions of PPTA-KOH-G, (c) N_2_ adsorption/desorption isotherms and (d) pore size distribution of PBO-KOH-G


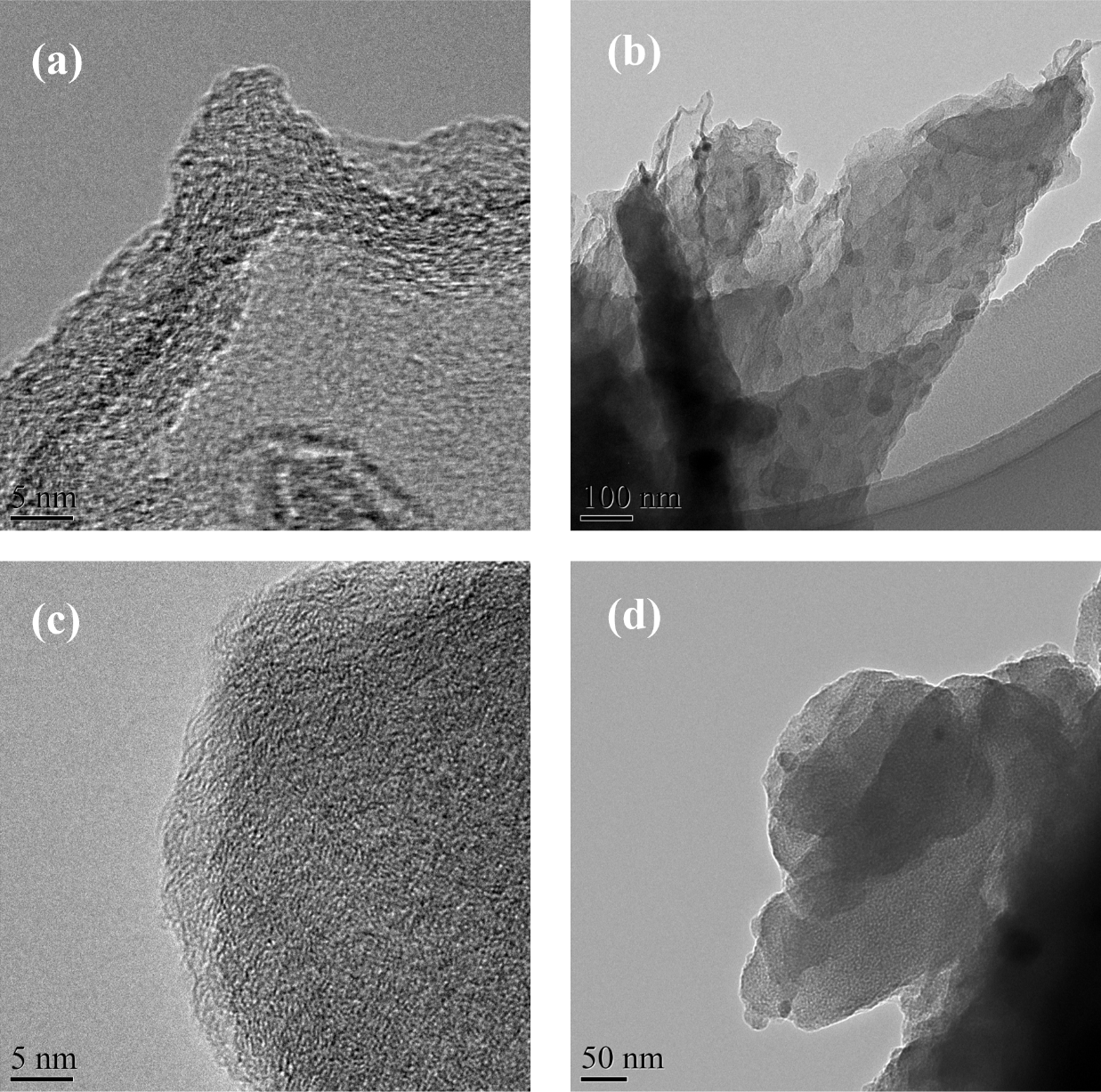


**Figure S4.** The TEM of (a) (b) PPTA-KOH-G and (c) (d) PBO-KOH-G.


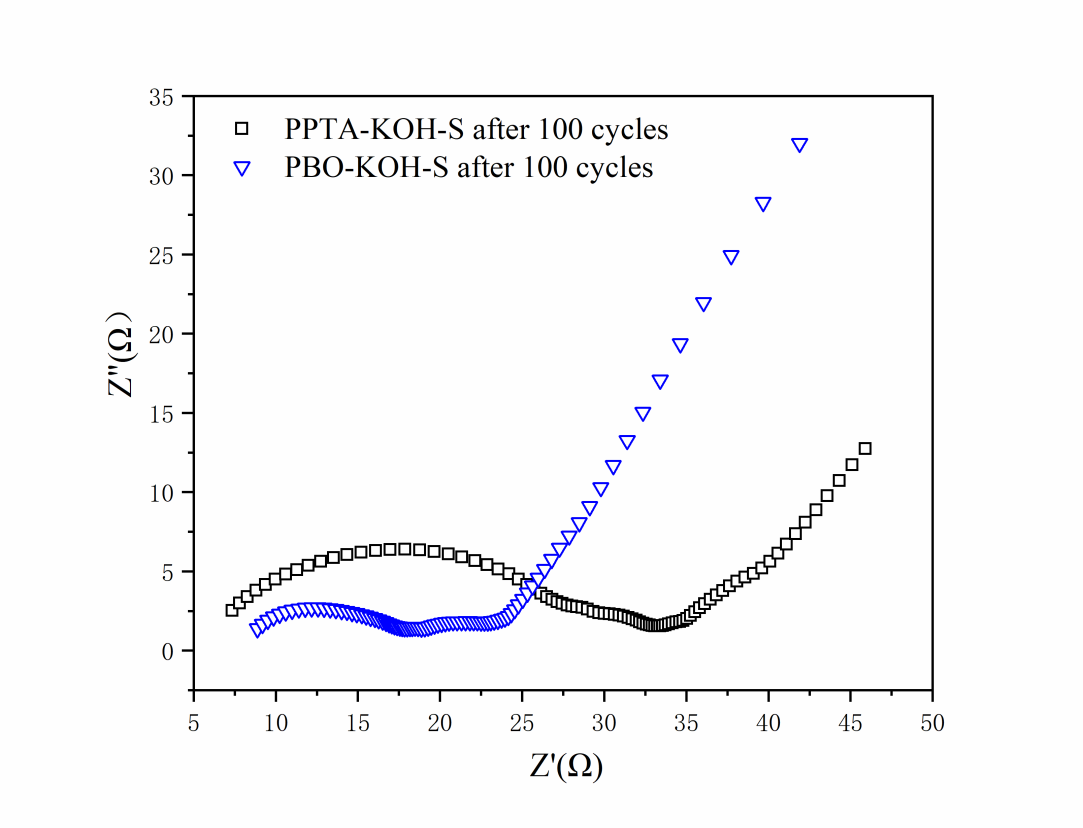


**Figure S5.** The EIS profiles of Li-S cells with PPTA-KOH-S and PBO-KOH-S as separators after 100 cycles and equivalent circuit models.


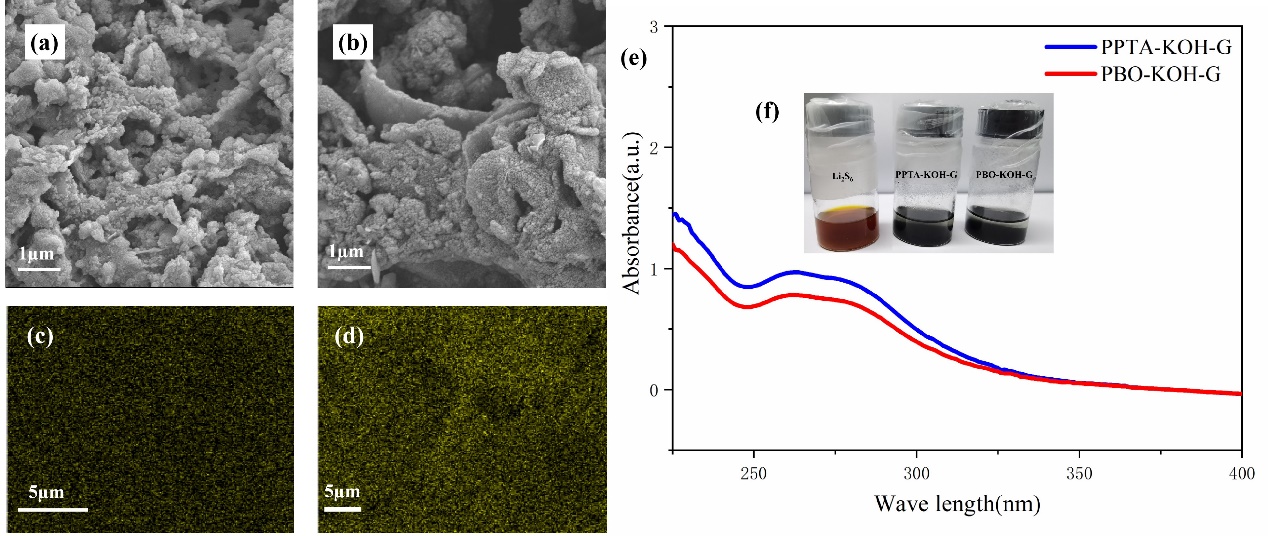
**Figure S6.** The SEM of (a) PPTA-KOH-S and (b) PBO-KOH-S after 200 cycles, the S of (c) PPTA-KOH-S and (d) PBO-KOH-S after 200 cycles, (e) ultraviolet-visible spectra of PPTA-KOH-G and PBO-KOH-G after adsorbing Li_2_S_6_ solution, (f) the visual adsorption experiment of PPTA-KOH-G and PBO-KOH-G


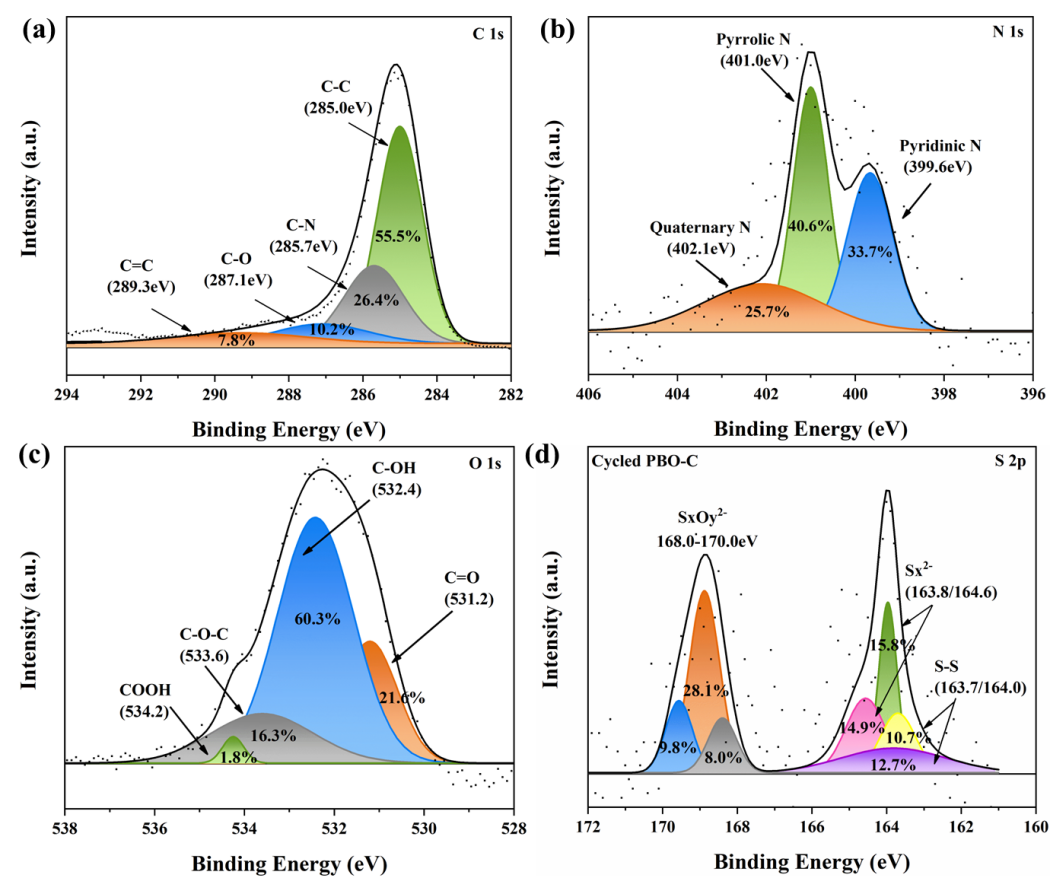


**Figure S7.** (a) C 1s, (b) N 1s, (c) O 1s, and (d) S2p XPS spectra of PBO-KOH-G after adsorption of polysulfide

**Table S1.** The weight percent of C, N, H and S element in PPTA-P、PPTA-KOH-G、PBO-P and PBO-KOH-G.

| **Samples** | **C (wt%)** | **N (wt%)** | **H (wt%)** | **S (wt%)** |
| --- | --- | --- | --- | --- |
| PPTA-P | 65.25 | 10.85 | 4.12 | 0 |
| PPTA-KOH-G | 59.52 | 4.74 | 2.79 | 0 |
| PBO-P | 68.37 | 10.52 | 3.72 | 0 |
| PBO-KOH-G | 58.22 | 5.50 | 2.60 | 0 |

**Table S2.** Pore analysis of PPTA and PBO based carbon materials.

| **Samples** | **Surface area (m^2^ g^-1^)** | **Median Pore size (nm)** | **Pore volume (cm^3^ g^-1^)** |
| --- | --- | --- | --- |
| PPTA-KOH-G | 1676.7 | 2.9 | 1.2 |
| PBO-KOH-G | 2104.4 | 2.6 | 1.3 |

**Table S3.** Summary of the electrochemical properties of Li-S batteries with various coating separators.

| **Coating separators** | **loading of sulfur**  **(mg cm^−2^)** | **Current** | **Capacity (mAh g^−1^)** | **Capacity**  **in cycles** | **Maximum C rate** | **Capacity (mAh g^−1^)** | **Ref** |
| --- | --- | --- | --- | --- | --- | --- | --- |
| Ce-MOF | 3.5 | 0.1C | 1366.3 | 889.2 after 100 cycles | 2C | 653.3 | [1] |
| rGO/Co-Ni-S composite | 2.0 | 1C | 1089 | 612 after 350 cycles | 8C | 610 | [2] |
| MoO_2_ nanosheets coupled with CNTs | 1.5 | 0.2C | 1375 | 738 after 300 cycles | 2C | 698 | [3] |
| Carbon Nanocages-Coated separators | 3.7 | 1C | 901 | 747 after 500 cycles | 3C | 639 | [4] |
| Cellulose fiber | 3.6 | 0.5C | 1016.0 | 599 after 800 cycles | 1.28C | 500 | [5] |
| A carbon mixed amorphous-TiSx | 2.8 | 0.1C | 1375 | 750 after 500 cycles | 4C | 356 | [6] |
| N, O co-doped chlorella-based biomass carbon | 1.5 to 2.0 | 0.2C | 1195 | 750 after 200 cycles | 2C | 902 | [7] |
| Co-N-C hollow nanocages | 3.0 | 0.5C | 822 | 725 after 200 cycles | 1C | 600 | [8] |
| TiO_2_ microbox carbon nanotube | 2.0 | 0.2C | 1800 | 852.3 after 200 cycles | 10C | 623 | [9] |
| ZIF-67/Super P modified separator | 1.3 | 0.2C | 1341 | 761 after 300 cycles | 2C | 751 | [10] |
| PPTA&PBO  carbon | 3.6 | 0.2C | 1321.6 | 827 after 200 cycles | 5C | 840.6 | Our work |

**Table S4.** Atomic compositions of the C、N、O and S measured by XPS survey results before and after cycled

| **Sample** | **C (at%)** | **O (at%)** | **N (at%)** | **S (at%)** |
| --- | --- | --- | --- | --- |
| PPTA-KOH-S | 77.9 | 8.44 | 4.22 | 0 |
| PBO-KOH-S | 72.06 | 9.29 | 5.64 | 0 |
| Cycled PPTA-KOH-S | 39.8 | 31.37 | 3.25 | 7.52 |
| Cycled PBO-KOH-S | 32.79 | 41.73 | 1.82 | 7.77 |

**References**

[1] Y. Su, W. Wang, W. Wang, A. Wang, Y. Huang, Y. Guan, *Journal of The Electrochemical Society*, 169 (**2022**).

[2] P. Wu, L. Tan, X.-D. Wang, P. Liao, Z. Liu, P.-P. Hou, Q.-Y. Zhou, X.-J. Jin, M.-C. Li, X.-R. Shao, Z. Zeng, S. Deng, G.-P. Dai, *Materials Research Bulletin*, 145 (**2022**).

[3] Z.-k. Kong, Y. Chen, J.-z. Hua, Y.-z. Zhang, L. Zhan, Y.-l. Wang, *New Carbon Materials*, 36 (**2021**) 810-820.

[4] Z. Yang, X. Zhang, Z. Li, L. Chen, Z. Song, J. Zhang, X. Qiu, Z. Zheng, *Energy & Fuels*, 35 (**2021**) 19843-19848.

[5] Y. Zhou, Y. Zhang, X. Li, *Materials Today Energy*, 19 (**2021**).

[6] Y. Yao, Y. Wu, N. Wang, M. Li, T. Hang, *Materials Chemistry and Physics*, 258 (**2021**).

[7] Q. Li, Y. Liu, L. Yang, Y. Wang, Y. Liu, Y. Chen, X. Guo, Z. Wu, B. Zhong, *Journal of Colloid and Interface Science*, 585 (**2021**) 43-50.

[8] L. Jin, Z. Fu, X. Qian, B. Huang, F. Li, Y. Wang, X. Shen, *Microporous and Mesoporous Materials*, 316 (**2021**).

[9] J. Ni, L. Jin, M. Xue, Q. Xiao, J. Zheng, J.P. Zheng, C. Zhang, *Journal of Solid State Electrochemistry*, 25 (**2020**) 949-961.

[10] B. Ma, Y. Gao, M. Niu, M. Luo, H. Li, Y. Bai, K. Sun, *Solid State Ionics*, 371 (**2021**).
